# Reproducing macaque lateral grasping and oculomotor networks using resting state functional connectivity and diffusion tractography

**DOI:** 10.1007/s00429-020-02142-2

**Published:** 2020-09-16

**Authors:** Henrietta Howells, Luciano Simone, Elena Borra, Luca Fornia, Gabriella Cerri, Giuseppe Luppino

**Affiliations:** 1 MoCA Laboratory, Department of Medical Biotechnology and Translational Medicine, University of Milan, Milan, Italy; 2 Department of Medicine and Surgery, Neuroscience Unit, University of Parma, Parma, Italy

**Keywords:** Comparative MRI, Macaque, Tractography, Resting state, Neural tracers, Motor control

## Abstract

Cortico-cortical networks involved in motor control have been well defined in the macaque using a range of invasive techniques. The advent of neuroimaging has enabled non-invasive study of these large-scale functionally specialized networks in the human brain; however, assessing its accuracy in reproducing genuine anatomy is more challenging. We set out to assess the similarities and differences between connections of macaque motor control networks defined using axonal tracing and those reproduced using structural and functional connectivity techniques. We processed a cohort of macaques scanned in vivo that were made available by the open access PRIME-DE resource, to evaluate connectivity using diffusion imaging tractography and resting state functional connectivity (rs-FC). Sectors of the lateral grasping and exploratory oculomotor networks were defined anatomically on structural images, and connections were reproduced using different structural and functional approaches (probabilistic and deterministic whole-brain and seed-based tractography; group template and native space functional connectivity analysis). The results showed that parieto-frontal connections were best reproduced using both structural and functional connectivity techniques. Tractography showed lower sensitivity but better specificity in reproducing connections identified by tracer data. Functional connectivity analysis performed in native space had higher sensitivity but lower specificity and was better at identifying connections between intrasulcal ROIs than group-level analysis. Connections of AIP were most consistently reproduced, although those connected with prefrontal sectors were not identified. We finally compared diffusion MR modelling with histology based on an injection in AIP and speculate on anatomical bases for the observed false negatives. Our results highlight the utility of precise ex vivo techniques to support the accuracy of neuroimaging in reproducing connections, which is relevant also for human studies.

**Electronic supplementary material:**

The online version of this article (10.1007/s00429-020-02142-2) contains supplementary material, which is available to authorized users.

## Introduction

One largely accepted current view in neuroscience is that cortical functions result from the integration of different, reciprocally connected areas working together as large-scale functionally specialised networks (for example, see Bressler and Menon 2010; Catani et al. 2012; Borra and Luppino 2019). Accordingly, to be able to fully understand cortical functions, definition of the underlying neural circuits connecting different structures and their functional interplay is necessarily required. To achieve this, experimental approaches are often combined in order to identify which cortical areas are involved in a given function and their possible reciprocal connectivity. By integrating these data, comprehensive models can be constructed of specialised circuits to identify the possible flows of information processing, and the neural mechanisms from which a given function can emerge.

These large-scale, functionally specialized networks have been carefully defined, in detail, for skilled motor control in the animal model (e.g., non-human primates) using the architectonic approach for the definition of cortical areas, single unit recording for their functional characterisation, and neural tracers to define their connectivity. For example, using this direct approach, different cortical sectors involved in distinct aspects of skilled hand actions have been identified and the circuits that connect them, recently formulated as the ‘lateral grasping network’ (Borra et al. 2017). The development of non-invasive functional and connectional imaging techniques has allowed for the construction of similar definitions of motor control pathways also in the human brain (Turella and Lingnau 2014), although these are still less clearly defined compared to evidence obtained in the macaque. Functionally distinct cortical sectors have been identified for highly skilled movement using intraoperative stimulation (Viganò et al. 2019; Fornia et al. 2020), and the connections have been studied using diffusion MRI tractography (dMRI) (Budisavljevic et al. 2016; Howells et al. 2018). Approaches measuring the spontaneous organized fluctuations of brain activity at rest (resting state functional connectivity; rs-FC) may also provide further evidence for large-scale, functionally specialized networks in the human brain (Simone et al. 2020), although the interpretation of these networks with respect to structural connections is still not clearly defined.

Diffusion MRI uses estimations of the orientation of diffusion of water molecules in tissue to reconstruct likely trajectories of white matter pathways (i.e., tractography), which are commonly used to construct whole brain structural ‘connectomes’ or visualisations of specific sets of connections using a ‘virtual dissection’ approach. This technique has rapidly become the most important tool for investigating connectional anatomy, however, comparisons with data from anatomical tracing in the macaque have questioned the technique’s validity for precise in vivo reproduction of sector-to-sector connectivity (Thomas et al. 2014; Reveley et al. 2015; Knösche et al. 2015; Jbabdi et al. 2015).

Resting state FC (rs-FC) measures temporal associations between brain regions based on spontaneous fluctuations of brain activity, measured with blood-oxygen-level-dependent (BOLD) signal. The definition of a ‘functional connection’ between two areas is, therefore, divergent between the rs-FC community and those that use electrophysiological approaches. Functional connectivity is related to, but distinct from, anatomical connectivity, as it may be subserved by both polysynaptic and monosynaptic anatomical circuits, and can be influenced by several factors in contrast to structural connectivity (Biswal et al. 2010; Buckner et al. 2013). Despite some limitations, these techniques provide a unique opportunity to acquire whole brain datasets that can be compared with human data, as well as between hemispheres in a single monkey (Croxson et al. 2018; Balezeau et al. 2020). Digital data also enable comparison of large groups of macaques within a single space (Thiebaut de Schotten et al. 2019). Furthermore, the use of non-invasive techniques in living monkeys provides more ethical, efficient data acquisition and enables longitudinal studies to be performed. As the field of comparative MRI grows, it is relevant to identify the false positives and negatives of different structural and functional connectivity neuroimaging techniques. The extent to which they provide us with comparable information to that provided by single unit recording, architectonics and tracing techniques still remains to be fully assessed.

In this context, one possible way for addressing this issue is to test the efficacy of these techniques in identifying large-scale motor control networks of the macaque brain that have been well defined based on neural tracers and electrophysiology. Non-human primate (NHP) imaging is still in its infancy in comparison with human imaging, as it requires custom-built equipment and sequences with high field strengths that require more complex processing pipelines. A valuable resource, PRIME-DE, has recently been created to pool international NHP neuroimaging data, with the intention of facilitating advances in this field and fostering collaborations (Milham et al. 2018; 2020). This resource provides structural, resting state and diffusion MRI data from over twenty sites worldwide as well as quality assessment of the provided datasets. In the present study, we used two macaque in vivo datasets provided by PRIME-DE to trace two, well-established large-scale networks of the macaque, each including specific sectors of the temporal, parietal, and frontal cortex, involved in controlling purposeful hand actions (“lateral grasping network” LGNet) and explorative oculomotor behaviour (“explorative oculomotor network” EONet) respectively (see Borra and Luppino 2019). To this aim, cortical sectors of these two networks were defined anatomically on structural images in every monkey and used as seeds for dMRI and rs-FC.

## Methods

### Data acquisition

Two in vivo cohorts of macaque monkeys were used for analysis (Mount Sinai: 9 datasets; UC Davis: 19 datasets; Table 1) for which resting state and diffusion imaging data had been acquired. These data were made available through PRIME-DE, an open NHP data sharing imaging resource (Milham et al. 2018). The UC Davis cohort was used to perform group and individual level rs-FC analysis, and the Mount Sinai cohort was used to perform individual level rs-FC analysis and diffusion tractography.

**Table 1:** Data on two macaque cohorts used in the present study

Dataset	Principal investigator	Number	Species	Mean age (years)	Weight (kg)	Housing	Sex
Mount Sinai	Paula Croxson	6	5 Macaca mulatta, 1 Macaca fascicularis	5	6.2	Groups of 6 (MF single)	5 M, 1 F
UC-Davis	Mark Baxter	19	Macaca mulatta	20.4	9.7	16 paired, 3 single	19 F

#### Mount Sinai data

The cohort consisted of nine datasets and we limited analysis to six macaques: five *Macaca mulatta* (male) and one *Macaca fascicularis* (female), that had undergone the diffusion imaging sequence (details at https://fcon_1000.projects.nitrc.org/indi/PRIME/mssm1.html). Ethical approval was provided by the Icahn School of Medicine at Mount Sinai Hospital (ISMMS) and supported by the Institutional Animal Care and Use Committee. The animals were anaesthetised with isoflurane (1.2%) and were monitored for the depth of anaesthesia (Froudist-Walsh et al. 2018). Monkeys were scanned on a 3T Philips Achieva scanner, with a four-channel phased array head coil (Windmiller-Kolster Scientific). Diffusion-weighted data were acquired with a 1 mm isotropic voxel size, collecting 120 directions (TE = 107 ms, TR = 11,000 ms), including 3 averages and reverse polarity images. A *b* value of 1000 s/mm^2^ was used with two interleaved b0s. T1 images were collected with an isotropic voxel resolution of 0.5 mm (TE = 6.93 ms, TR = 15 ms, TI = 1100 ms, flip angle 8 degrees).

#### UC-davis data

Nineteen macaque monkeys (*Macaca mulatta*) were included in this collection (details at https://fcon_1000.projects.nitrc.org/indi/PRIME/ucdavis.html). Ethical approval was provided by UC-Davis IACUC. All monkeys underwent neuroimaging under anaesthesia (isoflurane 1–2%) and no contrast agent was used. Monkeys were scanned on a 3T Siemens MAGNETOM Skyra scanner, fitted with a four-channel clamshell coil. Resting state data were collected using an isotropic voxel resolution of 1.4 mm to collect 250 volumes (TE = 24 ms, TR = 1600 ms), including a gradient echo image (acquisition time 6.67 min).

### Anatomical definition of LGNet and EONet nodes on structural images

The cortical sectors used as seeds for the whole-brain rs-FC and dMRI analyses were defined by two expert anatomists (E.B and G.L) on a high-quality template (INIA19) for non-human primate studies created from 100 high-resolution, *T*_1_-weighted magnetic resonance (MR) scans of 19 rhesus macaque (*Macaca mulatta*) animals (Rohlfing et al. 2012) (https://nitrc.org/projects/inia19/). The cortical sectors of the lateral grasping network (LGNet) and exploratory oculomotor network (EONet) were drawn by the authors on individual structural T1 images of each hemisphere of the six monkeys used from the Mount Sinai cohort in correspondence with the “core” of each cortical area, to exclude regions of possible transition between areas. Specific sectors (AIP, F5a, F5c, F5p, FEF, LIP, caudal and rostral TEa/m) were also drawn on the T1 of each of the 19 macaques in the UC Davis cohort. ROI sizes are provided in Supplementary Figure 1. The sectors (on the INIA and NMT templates) and the original table of ROI sizes are available to download from OSF (https://osf.io/5qamb/).

**Fig. 1:**
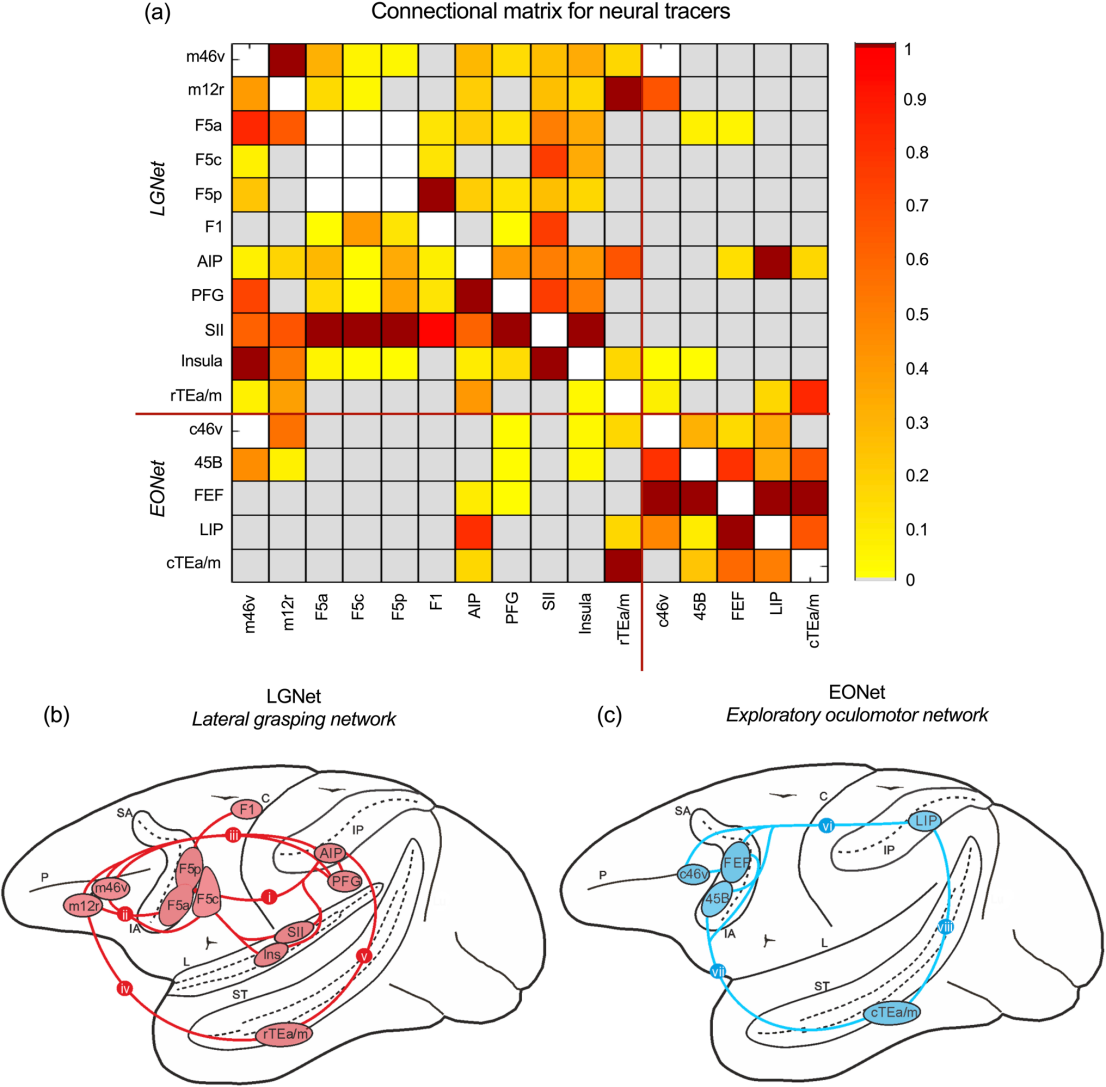
**a** The extent and strength of neural tracer connectivity between different sectors, amalgamated from previous studies (see text). The strength of the connections between areas of the two networks is normalized to the strongest one and indicated in dark red; **b** the LGNet (Borra and Luppino, 2017) in red and its (i) parieto-frontal, (ii) fronto-prefrontal, (iii) parieto-prefrontal, (iv) temporo-frontal, and (v) parieto-temporal connections; **c** The EONet in blue and its (vi) parieto-frontal, (vii) temporo-frontal, and (viii) parieto-temporal connections. *C* central sulcus, *IA* inferior arcuate sulcus, *IP* intraparietal sulcus, *L* lateral fissure, *P* principal sulcus, *SA* superior arcuate sulcus, *ST* superior temporal sulcus

The sectors that were the object of the present study were localised using anatomical landmarks and stereotactic coordinates based on architectonic and/or connectional criteria described in previous studies (Fig. 1a). Specifically, prefrontal areas 8/FEF, 45B, the middle part of area 12r (m12r), and the middle (m46v) and caudal (c46v) part of area 46v were defined based on architectonic (Gerbella et al. 2007) and connectional (Borra et al. 2010; Gerbella et al. 2010, 2013) studies. Ventral premotor area F5 subdivisions (F5a, F5p, F5c) were defined based on the architectonic study of (Belmalih et al. 2009) and the primary motor area F1 sector connected to F5p based on connectional criteria (Borra et al. 2010; Gerbella et al. 2011). The inferior parietal area PFG and the intraparietal area AIP were defined based on architectonic (Gregoriou et al. 2006) and connectional (Borra et al. 2008) data. The intraparietal area LIP was defined as a cortical sector located just caudal to AIP and extending 6 mm in the AP direction (Blatt et al. 1990). The insular, SII, and the rostral TEa/m sectors involved in the lateral grasping network were defined based on connectional criteria, using the maps shown in Borra and Luppino (2017). The caudal TEa/m sector, involved in the oculomotor network, was defined as a cortical sector located just caudal to the rostral, hand-related, one and extending 5 mm in the AP direction, based on connectional data (see, e.g., Cavada and Goldman-Rakic 1989; Blatt et al. 1990; Gerbella et al. 2010).

#### Tract tracing connectional matrix of the LGNet and EONet

To assess the strength of the connections between the various areas of the two networks under study, a connectional matrix was built based mostly on quantitative data available from previous studies (Rozzi et al. 2006; Borra et al. 2008, 2011; Gerbella et al. 2010, 2011, 2013). In most of these studies, quantitative data were typically analysed in terms of percent areal distribution of the labelled cells after a tracer injection in a given area. For the purposes of the present study, we considered and normalized data on the connections of a given area only with the core areas of the two large-scale networks under study. For area LIP, the hand-related sectors of F1, SII and the insula, and for the two area TEa/m sectors, the connectional strength was estimated, when possible, based on evaluation of the description of the data provided in studies in which neural tracers have been injected in these areas (Mesulam and Mufson 1982; Matelli et al. 1986; Cavada and Goldman-Rakic 1989; Blatt et al. 1990; Cipolloni and Pandya 1999; Jezzini et al. 2015) and/or indirect data from tracer injections placed in other areas of the two networks.

### Processing and analysis of structural imaging data

#### Preprocessing

Raw diffusion weighted imaging data were first corrected for noise (Veraart et al. 2016) and Gibbs ringing (Kellner et al. 2016) artefacts using MRtrix (Tournier et al. 2019). Diffusion images were then corrected for motion and eddy current distortions with ExploreDTI, regularised for low SNR data and registered to the 1 mm downsampled structural T1 using cubic spline interpolation.

The data were processed using High Angular Resolution Diffusion Imaging (HARDI) spherical deconvolution with a damped Richardson-Lucy algorithm to model the fibre orientation distribution function (fODF) within each voxel, using StarTrack (www.mr-startrack.com). This approach models multiple complex fibre orientations within a single voxel as opposed to diffusion tensor approaches (Dell’Acqua and Tournier 2018).

Parameters used for modelling were calibrated in StarTrack to optimise the fODF. Each dataset was processed using whole-brain probabilistic, ROI-to-ROI probabilistic and whole-brain deterministic tractography for comparison of the methods. An α value of 1.7, 600 iterations, an η of 0.001 and an r of 5 (Dell’Acqua et al. 2013). For both whole-brain and ROI-based probabilistic tracking an absolute threshold of 0.003 was used with an angle threshold of 20° and 20 bootstraps. For deterministic tractography, an absolute threshold of 0.001 was used with an angle threshold of 35°. The step size for all tracking was 0.5 mm and was limited to streamlines between 10 and 150 mm.

#### Virtual tract dissection

For whole brain tracking, a two-ROI approach was used to show streamlines passing between different sectors, which were extended slightly into white matter (Supplementary Figure 2; Thiebaut de Schotten et al. 2011a).

For each monkey, the number of streamlines and total voxels occupying the connection were extracted for the connections extending between each pair of ROIs of the LGN and EON. This was performed in each hemisphere using TrackVis software for each of the three tractography approaches. The highest value between hemispheres was used to assess strength of connectivity.

Comparisons between tracking approaches were performed using Spearman’s rank correlation co-efficients. If over half of monkeys showed the presence of a connection using one of the three tractography methods, this was classified as structurally connected. If over half of monkeys showed the presence of a connection based on resting state fMRI, this was classified as functionally connected. These values were used to calculate ROC curves using SPSS (v.26) to compare with the tracing data.

### Processing and analysis of functional imaging data

#### Image preprocessing

Preprocessing of the resting state fMRI data was conducted using Statistical Parametric Mapping 12 (SPM12) software (https://www.fil.ion.ucl.ac.uk/spm/). We performed two preprocessing pipelines based on the subsequent analysis to perform. Both Mount Sinai and UC Davis datasets were preprocessed for individual level analysis performed in native space, including slice-timing correction, rigid-body correction for head motion, rigid-body co-registration of the fMRI volumes with the high-resolution T1-weighted structural image and functional smoothing with a Gaussian kernel of 2 mm^3^ full-width half-maximum.

The UC Davis dataset was also preprocessed to perform a second level analysis (group analysis). Specifically, the preprocessing included: slice timing correction, rigid-body correction for head motion, functional outlier detection for scrubbing, co-registration of the fMRI volumes with the high-resolution T1-weighted structural image, cortical segmentations of the T1 image, spatial normalization of functional volumes and functional smoothing with a Gaussian kernel of 2 mm^3^ full-width half-maximum. Structural segmentation and spatial normalization were performed to match the INIA template brain (Rohlfing et al. 2012).

#### Resting state analysis

Seed-based resting state analyses were computed to evaluate the interactions of each ROI with other sectors using the Functional Connectivity (CONN) toolbox (https://www.nitrc.org/projects/conn), a MATLAB/SPM-based cross-platform open-source software. After preprocessing, images were band-pass filtered to 0.008–0.09 Hz and motion regressed to diminish the impact of noise.

ROI-to-ROI resting state analysis was computed by calculating the temporal correlation between the average BOLD signals from a given ROI to all other ROIs in the brain. Fisher Z-transformation was applied to correlation maps to achieve normality. ROI-based functional connectivity of each monkey in the UC Davis dataset was calculated using a general linear model, to determine whole brain resting state ROI correlations on the individual level (within subject, 1st level analysis). For each source ROI, for each monkey, first level results consisted of ROI-to-ROI functional connectivity maps. The connectivity maps were then entered into a second level general linear model to obtain population-level estimates. We used an uncorrected *p* value height threshold of < 0.001, with a cluster threshold of *p* < 0.05 (cluster-size p-FDR corrected) as the extent threshold for the whole brain. Finally, the significant differences in functional connectivity patterns between two different seeds was estimated by means of two paired *t* tests for “between-source” differences (FWE p-FWE < 0.05 cluster-corrected, *p* < 0.05).

Second level analysis was performed only using those sectors that showed positive correlations. Since paired *t* tests were performed to show only those areas showing higher connectivity, regions showing negative correlations were excluded. To this end, an explicit mask was used defined by those voxels that in the functional maps showed positive correlations.

## Results

### The lateral grasping and the explorative oculomotor network of the macaque brain

In the present study, we sought to trace, using MR-based approaches, two large-scale functionally specialized cortical motor control networks—the lateral grasping network (LGNet) and exploratory oculomotor network (EONet)—defined in the macaque brain based on connectional and functional data. To obtain a framework of reference for comparing tract tracing with dMRI and rs-FC data, we characterized the strength of connections between various cortical sectors, based on previous studies evaluating the areal distribution of labelled cells after a tracer injection in a given area (Fig. 1a).

The LGNet is made up of interconnected parietal, temporal and frontal areas considered to play a crucial role in controlling purposeful hand actions and hand action observation (see, for a detailed description and references, Borra et al. 2017; Fig. 1b). This network is centred on the robust and reciprocal connections of the various subdivisions of the hand-related ventral premotor (PMv) area F5 with the hand-related IPL areas AIP and PFG and with the hand field of the opercular parietal area SII (Fig. 1b—i). These connections mediate visuo- and somato-motor transformations which result in the activation of specific hand actions motor programs based on information on the object’s properties. The PMv and IPL areas of the LGNet are connected to specific sectors of the ventrolateral prefrontal cortex (VLPF): m46v and m12r (Fig. 1b—i, iii). These two prefrontal sectors could play a role in selecting appropriate hand motor programs based on contextual information, behavioural goals and guiding rules and current, memorized, or working memory information on object properties and motor programs. A further node of the LGNet is a relatively rostral sector of the inferotemporal area TEa/m (rTEa/m) that is connected to both areas AIP and m12r (Fig. 1b—iv, v). This node, which is part of the ventral visual stream, could play a role in the selection of hand motor programs based on the identity of the object target of the action. Finally, a hand-related sector of the insular cortex is connected to IPL, PMv, and VLPF nodes of the LGNet and is a possible source of signals related to internal states modulating the control of hand actions. The contribution of other areas connected to nodes of the LGNet, such as, for example, F6/pre-SMA, have not been considered in the present study.

The EONet is a network of interconnected parietal, temporal and frontal areas, which could play a crucial role in guiding oculomotor behaviour for the exploration of visual scenes and perception of objects, actions, and faces (Fig. 1c; see, for a detailed description and for references, Borra and Luppino 2019). This network is centred on a parietofrontal circuit linking visually responsive oculomotor areas: the lateral intraparietal (LIP) area and two frontal areas, the frontal eye field (FEF) and area 45B (Fig. 1c—vi). This circuitry plays a crucial role in visuomotor transformations for controlling saccadic eye movements and in the orientation of spatial attention. Both frontal and parietal nodes of the EONet are connected to c46v and to a TEa/m sector located just caudal to the sector involved in the LGNet (cTEa/m) (Fig. 1c vii, viii). This inferotemporal sector is part of the ventral visual stream component specifically dedicated to 3D object and action processing. Accordingly, there is evidence for a large-scale temporo-parieto-frontal network where visuospatial dorsal visual stream information and ventral visual stream information on objects and actions could be used for guiding small-amplitude saccades. The EONet also includes the supplementary eye field, located in the dorsal premotor cortex, not considered in the present study.

### Connectivity of the LGNet and EONet in the Mount Sinai cohort

#### Connections of the LGNet

**Fig. 2:**
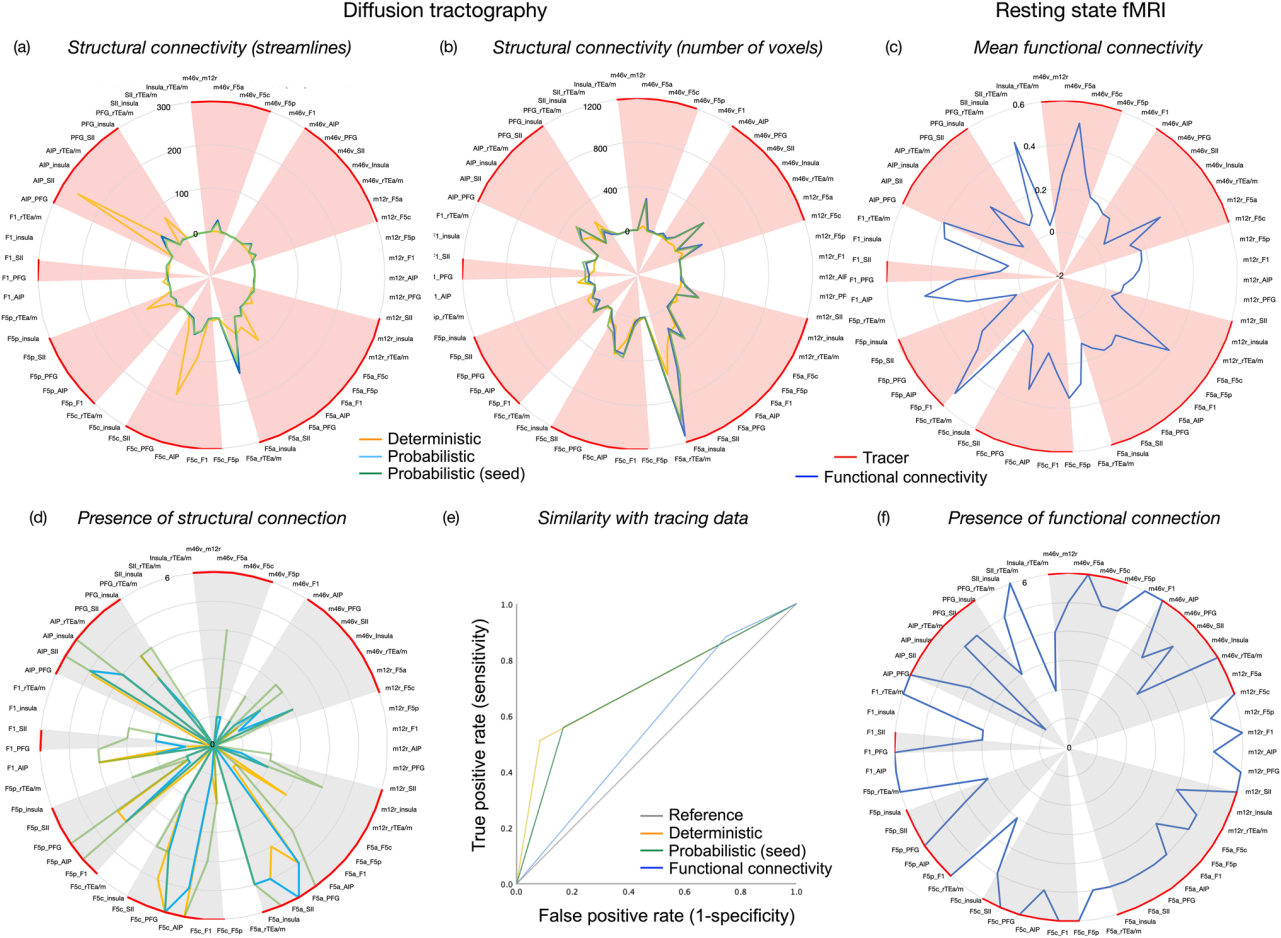
Connections of the LGNet shown as radar charts, based on **a** the average number of streamlines across the six monkeys, **b** the average number of voxels occupied by these streamlines across the six monkeys **c** mean functional connectivity, based on the average computed on the individual level, and mean structural connectivity for each measure across six monkeys (converted to standard scores). The consistency of these connections across the six monkeys are shown as radar charts for **d** structural connectivity and (f) functional connectivity. The shaded region (red, grey) indicates that a connection has been identified using tracing **e** A ROC curve shows the sensitivity and specificity of each structural and functional imaging technique to identify connections revealed by tracers

Connections between each pair of ROIs was calculated and compared across the three tractography approaches. Mean connectivity (streamline count and number of voxels) was highly correlated between ROI-to-ROI and whole brain probabilistic tracking approaches (*r* = 0.991, *p* < 0.001; *r* = 0.993; *p* < 0.001; Fig. 2a), although the incidence of connections across monkeys was higher when using a ROI-to-ROI probabilistic approach (Fig. 2d). Both deterministic and probabilistic tractography methods identified the greatest number of streamlines between frontal and parietal sectors (e.g. AIP- SII, F5c-PFG, AIP-F5c). Probabilistic methods were better able to identify streamlines connecting different frontal regions (e.g., F5a-m46v, m46v-insula, m12r-F5a). Connections were most commonly identified across monkeys between frontal and parietal sectors, irrespective of the tracking method (in particular F5c-AIP, F5c-PFG, F5a-insula, F5a-SII, F5a-PFG).

Deterministic tracking showed 18 false negative connections, and 1 false positive (F1-AIP). Whole brain probabilistic tracking showed 16 false negative connections, and 2 false positives (F1-AIP and F1-insula). ROI-to-ROI-based tracking showed 13 false negative connections and 3 false positives (m12r-PFG, F1-AIP and F1-insula).

A ROC curve showed that deterministic tracking had a true positive rate just over chance (51%) and a false positive rate of 8% (Fig. 2e). For probabilistic tracking, there was a true positive rate of 56% and a false positive rate of 16%. Hence while deterministic tracking is less sensitive than probabilistic tracking in identifying connections, it had slightly higher specificity. The area under the ROC curve was 0.71 for deterministic tracking and 0.69 for probabilistic tracking. This indicates that both methods are reasonably good at discriminating the presence of a connection identified with tracers.

Functional connectivity between each pair of sectors was computed on the individual level for each of the six monkeys from Mount Sinai dataset. Figure 2c shows the average rs-FC across the six monkeys. Connections between most ROIs could be identified, but with a high rate of false negatives. Notably, these connections were identified more consistently across monkeys (Fig. 2f).

When comparing whether the connectivity between sectors based on functional connectivity was comparable with those identified with tracers, a ROC curve showed a true positive rate of 88% and false positive rate of 75% (Fig. 2e). This indicated that rs-FC was more sensitive in identifying connections shown by tracers than diffusion tractography, but less specific. The area under the ROC curve was 0.56, indicating this approach discriminates connections identified with tracers at just above chance level.

#### Connections of the EONet

**Fig. 3:**
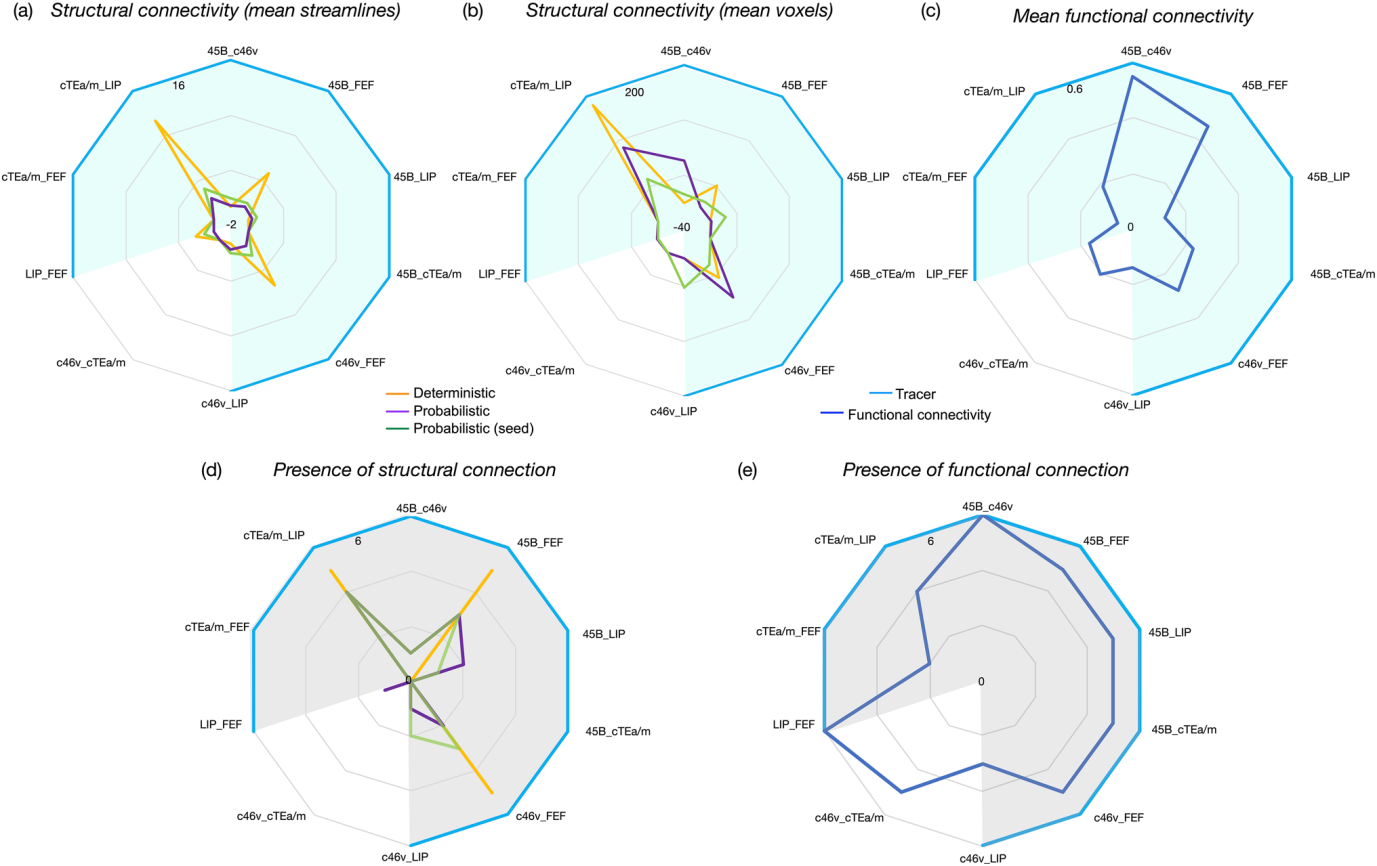
Connections of the EONet shown as radar charts, based on **a** the average number of streamlines, **b** the average number of voxels occupied by these streamlines and **c** mean functional connectivity across the six monkeys. The consistency of these connections across the six monkeys are shown as radar charts for **d** structural connectivity and **e** functional connectivity. The shaded region (blue, grey) indicates that a connection was identified using tracing

Structural approaches primarily identified connections between LIP-cTEa/m, 45B-FEF and c46v-FEF. The mean number of streamlines was highest for these three sets of connections and could be identified in over half of monkeys in all cases (Fig. 3a, d). Deterministic tracking methods could identify the most streamlines in the greatest number of monkeys. Probabilistic tracking methods showed much fewer sets of streamlines between these sectors. Functional connectivity was present between EONet sectors in most monkeys, but this was relatively low. The highest rs-FC between sectors was identified between neighbouring prefrontal areas 45B-c46v and 45B-FEF (Fig. 3c).

Structural connectivity methods identified no false positives although only three connections were detected of a possible nine that had been identified with tracers. Weak functional connectivity was identified between 46v and cTEa/m, which is also weakly identifiable when using tracers. Due to the low number of connections, we did not calculate ROC curves.

### Functional connectivity of the LGNet and EONet in the UC Davis cohort

#### Seed based functional connectivity between sectors (group level analysis)

**Fig. 4:**
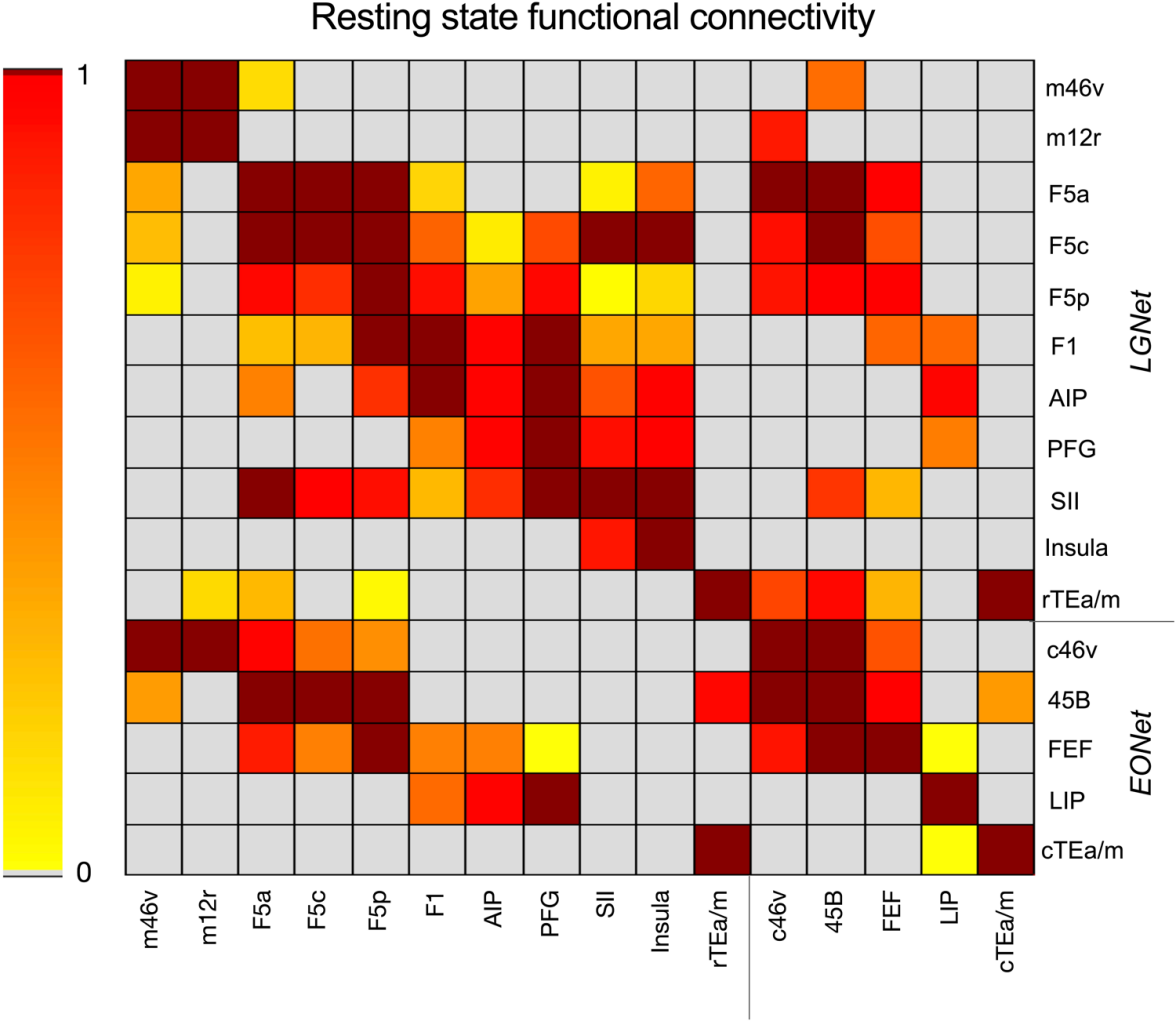
Correlation matrix showing the functional connectivity group analysis performed in the UC Davis cohort. Each pairwise functional connectivity is represented by the number of significant voxels measured in the target ROI divided by the total number of voxels of the same ROI. Only the seed ROIs of the right hemisphere are represented, ROIs are listed according to their anatomical order within the LGNet and EONet

Figure 4 shows the results of the ROI-to-ROI analysis carried out on the group level using the UC Davis dataset. Each functional connection is represented by the ratio of the number of significant voxels found in the target ROI with the total number of voxels of the target ROI. Only those voxels showing a *Z* score > 2.3 were considered. In both rows and columns, the ROIs are listed according to their anatomical location from anterior to posterior within the LGNet and EONet. The matrix shows functional connectivity between regions in the right hemisphere.

The upper left part of the matrix (Fig. 4) shows the ROI-to-ROI rs-FC of the regions embedded in the LGNet (m46v, m12r, F5a, F5c, F5p, F1, AIP, PFG, SII, insula and rTEa/m). Seeding from hand-related prefrontal regions m46v and m12r showed these were functionally correlated with each other, while only the former was functionally connected with all the target premotor F5 sectors. Premotor sectors were strongly functionally connected to each other, as well as with F1, and F5p showed the strongest functional connection. When seeding from AIP, PFG and SII, these regions were primarily functionally coupled with other parietal sectors. Furthermore, while the SII seed was functionally connected with all F5 subsectors, F1 and the insula, seeding from AIP showed functionally connections only with F5a, F5p and F1. Using PFG as a seed, there was functional coupling with F1 only. When the rostral sector of TEa/m within the hand-related network was used as a seed, weak functional coupling was identified with m12r and with F5p and F5a.

The lower right part of the matrix (Fig. 4) shows the ROI-to-ROI rs-FC of the regions embedded in the EONet (c46v, 45B, FEF, LIP, and cTEa/m). The prefrontal sectors c46v, 45B and FEF formed a functional network. The parietal region LIP was not functionally connected with any regions embedded in the EONet, while the cTEa/m sector was weakly functionally connected with the target region 45B. The matrix also shows functional connectivity between regions belonging to different anatomical networks. Specifically, all F5 sectors and rTEa/m (part of the LGNet), were functionally coupled with the target prefrontal regions (c46v, 45B and FEF) involved in oculomotor behaviour (EONet). When using FEF and LIP as seeds, these were functionally connected with F1.

#### Comparison of functional connectivity of adjacent LGNet and EONet nodes

**Fig. 5:**
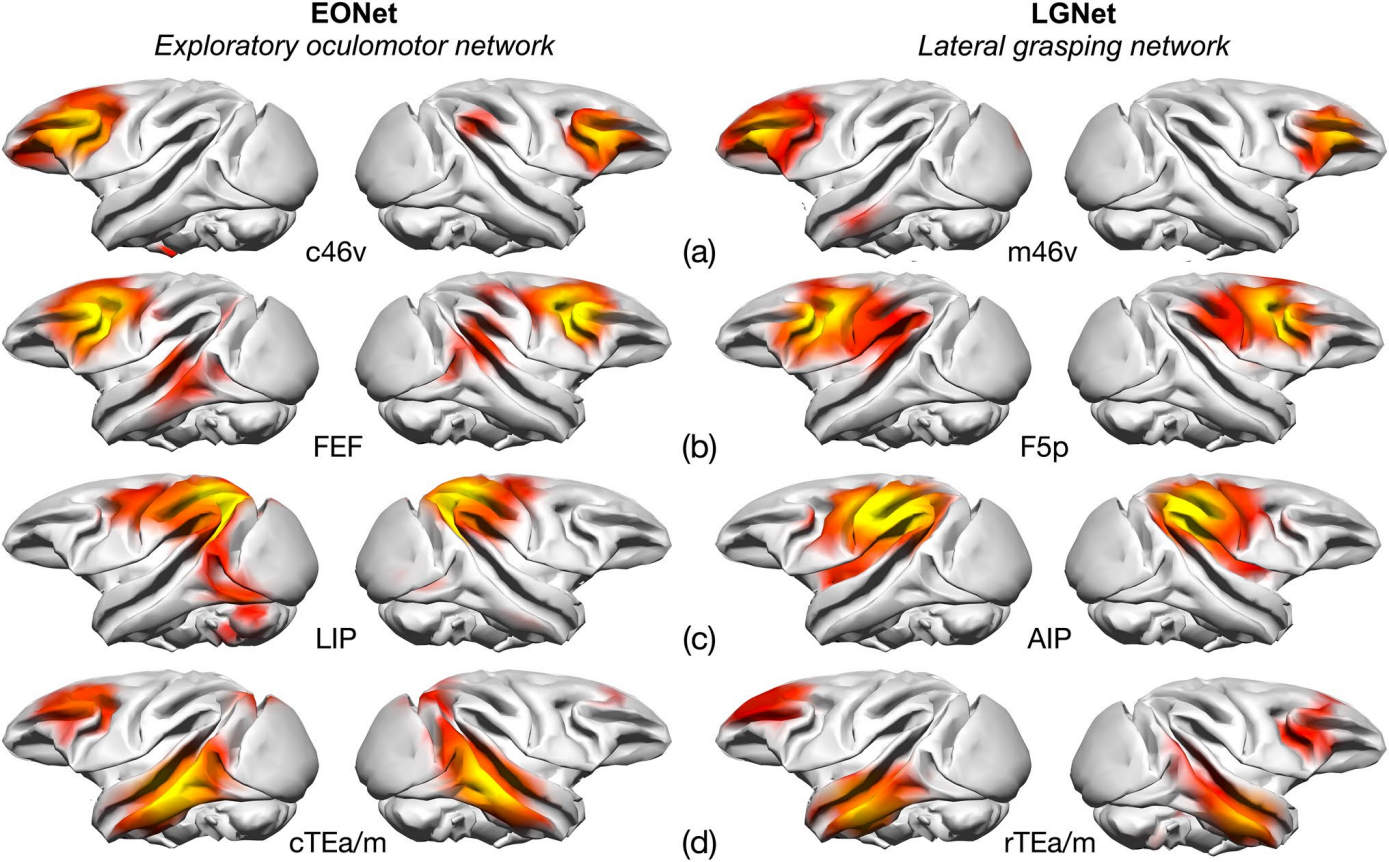
Comparison of the functional networks obtained by seeding neighbouring prefrontal, premotor, parietal and temporal regions shown on the INIA template, embedded in the EONet (left side) and LGNet (right side). These are compared in the text between **a** c46v and m46v **b** FEF and F5p **c** LIP and AIP and **d** cTEa/m and rTEa/m. Each hemisphere shows the rs-FC of the seed located within the same hemisphere

We performed two-paired t tests to identify “between-source” differences (cluster-size p-FWE corrected, *p* < 0.05, height threshold *p* < 0.001 uncorrected) to assess whether hand- (LGNet) and eye- (EONet) related neighbouring areas exhibited different functional connectivity patterns (Fig. 5). We compared the functional connectivity of eye-related sectors c46v, 45B, FEF, LIP and cTEa/m) with those of the respectively adjacent hand-related areas m46v, F5a, F5p, AIP and rTEa/m. The results showed that in six out of ten comparisons (m46v > c46v, c46v > m46v, F5a > 45B, 45B > F5a, F5p > FEF, FEF > F5p, AIP > LIP, LIP > AIP, rTEa/m > cTEa/m, cTEa/m > rTEa/m) functional connectivity was stronger only in the immediate proximity of the reference area (intraregional connectivity). The other four comparisons revealed that 45B had stronger rs-FC with c46v and rTEa/m, compared to F5a. F5p was more strongly functionally connected with F1 and the insula compared to the FEF (Fig. 5b). AIP was more functionally connected with F1, SII and the insula with respect to LIP (Fig. 5c) and finally the rs-FC of rTEa/m was higher with F5a and 45B compared to that of cTEa/m (Fig. 5d).

#### Seed-based functional connectivity between sectors (single level analysis)

Although the parieto-frontal rs-FC obtained through seeding anatomically defined intraparietal regions AIP and LIP largely overlapped with those obtained by spherical regions located in the same cortical position (Mars et al. 2011), the group level analysis did not identify rs-FC between AIP and LIP with F5c and FEF, respectively. Given that these functional connections were identified when the frontal regions were defined as seeds and the parietal ones as target (see F5c-AIP and FEF-LIP in Fig. 4) such a discrepancy may have resulted from inter-individual differences in areal localization of intrasulcal areas. We performed first level ROI-to-ROI analysis in the 19 monkeys of the UC Davis dataset in native space to evaluate whether inter-individual differences in areal localization could affect the definition of functional networks identified in the group analysis. To this end, specific ROIs of each network (F5a, F5c, F5p, FEF, AIP, LIP, caudal TEa/m and rostral TEa/m) were identified on the individual level in the right hemisphere. For each pair, we calculated the ratio between the number of significant voxels measured in the target ROI (*Z* score > 2.3) divided by the total number of voxels of the same ROI. Our results showed that, in spite of a higher degree of variability among individuals, we were able to identify the parieto-frontal connectivity between all F5 subsectors with AIP and between FEF and LIP. On average, seeding from F5p yielded functional correlations with 48% of AIP voxels, while seeding from F5a and F5c showed functional connections with 43% and 32% of AIP voxels, respectively. On average, seeding from FEF revealed functional correlations with 36% of LIP voxels. A lower percentage of significant voxels were identified, on average, in these intraparietal regions, by seeding from both the caudal and the rostral temporal sectors (caudal TEa/m-AIP 14%, caudal TEa/m-LIP 27%, rostral TEa/m-AIP 26%, rostral TEa/m-LIP 24%). Thus, parieto-frontal rs-FC was stronger than parieto-temporal and temporo-frontal rs-FC (rostral TEa/m-F5a 33%, rostral TEa/m-FEF 36%, caudal TEa/m- FEF 25%).

### Comparison with tracing data

**Fig. 6:**
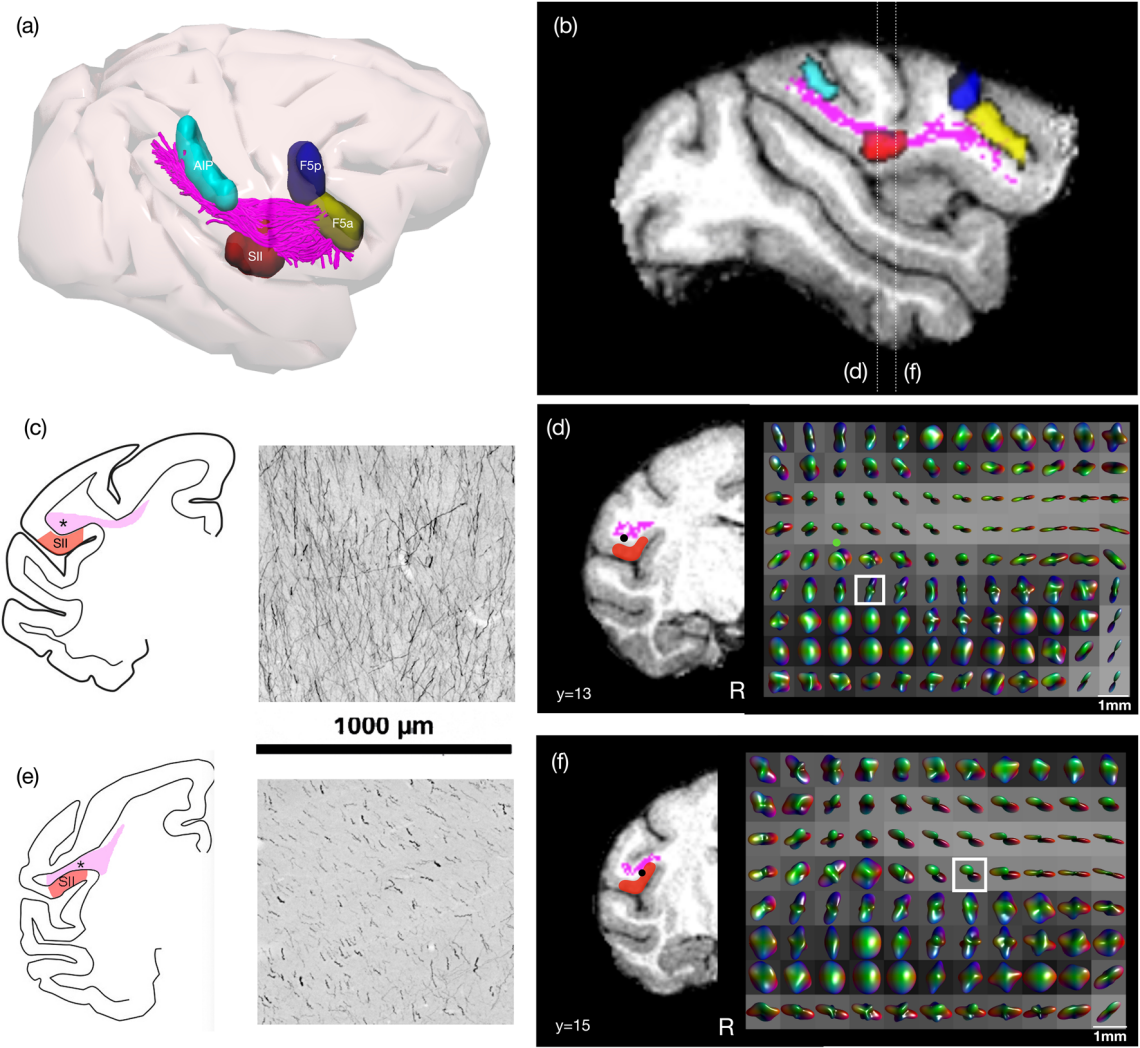
Comparison of labelled axons extending from the intraparietal area AIP in a macaque from a previous study (Case 30, Borra et al. 2008), with tractography and fibre ODF from spherical deconvolution modelling in one macaque from the Mount Sinai dataset. **a** Tractography of parieto-frontal connections of the LGNet running from AIP shown in 3D with respective sectors and **b** a sagittal slice showing this as a section. Comparison of **c** orderly axons within AIP-frontal projections running into SII which is also possibly reflected in a similar slice taken from the diffusion MR showing **d** fibre ODFs. **e** Orderly axons running anterior–posterior in a comparable section running within the core of the AIP-frontal projections and **f** fibre ODFs in a comparable coronal slice using diffusion MR. (N.B. in **e** there is also some cortical labelling)

**Fig. 7:**
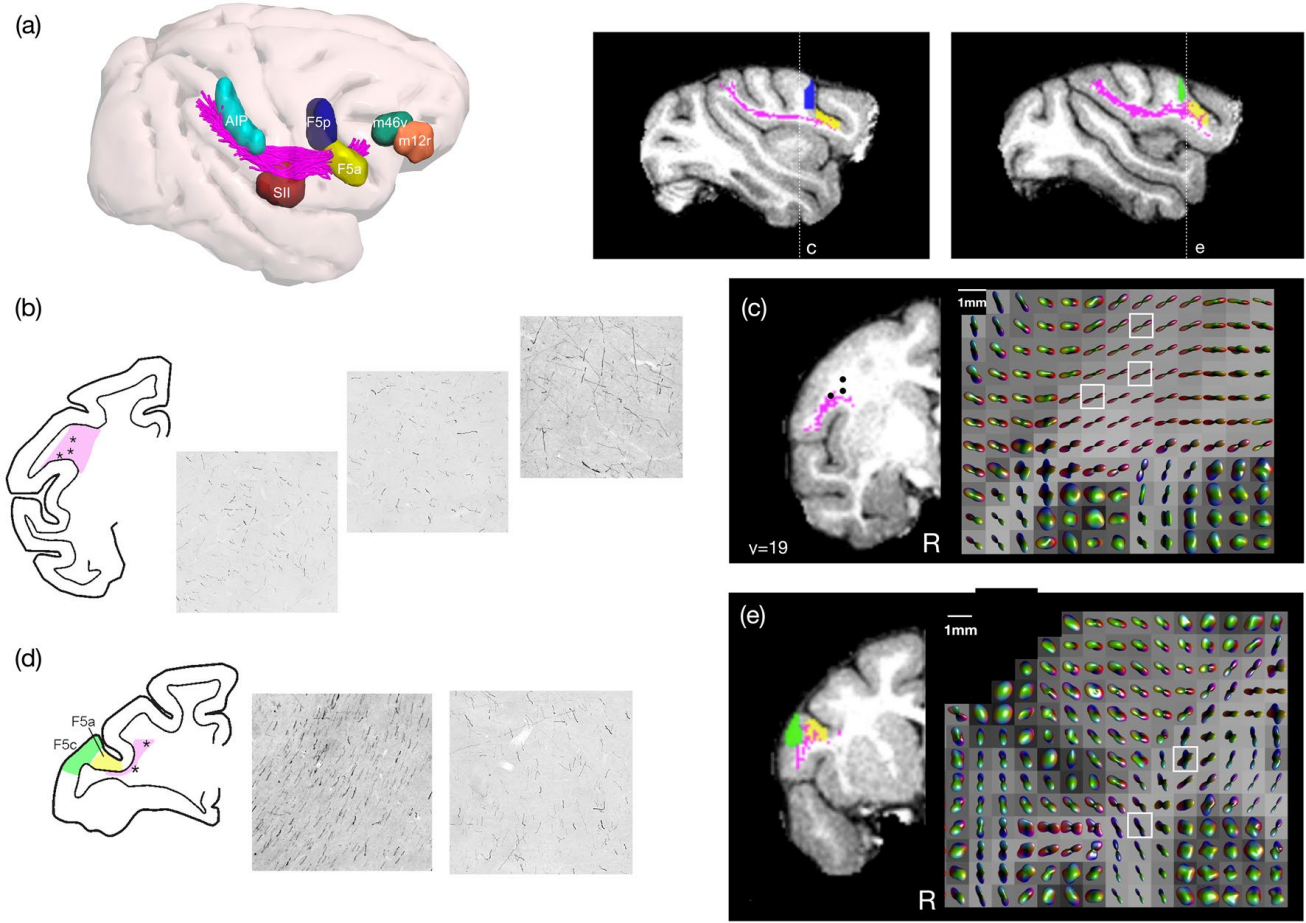
Frontal lobe comparison of labelled axons extending from AIP from the same case shown in Fig. 6, **a** tractography and fibre ODF from spherical deconvolution modelling in one macaque from the Mount Sinai dataset, **b**, **c** in a coronal slice caudal to F5p and **d**, **e** a coronal slice close to F5a and F5c. In representative samples **b** AIP-F5 axons caudal and medial to F5p project in different directions while in the more lateral caudal sites they are packed and parallel. The white matter section was obtained just behind layer 6 of F5p. **d** a representative section shows labelled axons extending beneath the arcuate sulcus (yellow), as well as other fibres running anterior–posterior (green). This may also be reflected in **e** fODF generated in comparable coronal slices using dMRI output

The most consistent connections identified with both structural and functional connectivity techniques were those running between frontal and parietal regions. Hence, we compared the results from an injection of an anterograde neural tracer placed in the centre of AIP (biotinylated dextran amine, BDA, Case 30 in Borra et al. 2008) showing labelled axons originating from the entire dorso-ventral extent of a sector of this area extending for 2 mm in rostrocaudal direction with the fibre orientation distribution function (fODF) generated using spherical deconvolution modelling and the tractography generated from this output (Figs. 6 and 7).

Tractography could reproduce connections between AIP and different F5 sectors, although no inference as to directionality can be made using this technique (Fig. 2). Labelled axons projecting from AIP also run into SII as well as toward F5 and more prefrontal sectors. The example shown here indicates that within a region equivalent to a single voxel, sets of axons can be conserved in their ventral trajectory into SII (Fig. 6c). When evaluating the fODF used to generate tractography, representative voxels may show similar variation in projections within the region of the AIP-F5 connection running dorsal to SII (Fig. 6d). In fact, structural connections of AIP were most commonly identified with SII (Fig. 2a, e). In certain sites (Fig. 6e), a representative section of labelled axons reflects a posterior-to-anterior trajectory, which may also be shown in corresponding voxels modelled with fODFs (Fig. 6f).

In the frontal lobe, labelled axons from AIP extend through and beneath F5a, running below the tip of the inferior arcuate sulcus. These fibres were also commonly reproduced using tractography (Fig. 2a). Axons then extend rostral and dorsally to extend into areas 46v and 12r: this portion of the tract was not reproduced using tractography. Just caudal to F5p and the inferior arcuate bank, coronal sections show many different crossing fibres of axons running within different portions of the parieto-frontal bundle, which may reflect projections to different cortical sectors (Fig. 7b). Very few fibres could be reproduced connecting AIP and F5p using tractography, although these were identified in all monkeys (Fig. 2a, d), which we speculate may be due to the more complex fibre composition in this region. This is echoed in maps of the fODF used for generating streamlines; certain voxels are more isotropic, which may reflect multiple fibre trajectories within these voxels.

This may be particularly relevant for projections extending beyond F5a toward prefrontal sectors. At this stage, tractography reconstructions show streamlines pass through F5a (Fig. 7a); however, these appear to terminate before reaching prefrontal sectors, which is supported by the results in Fig. 2 showing very few connections identified between AIP and prefrontal sectors. When comparing sections of labelled axons running from AIP with fODFs generated in a representative slice, axon populations running from AIP differ in density in different portions of the tract—medially, these fibres are less densely organised, while more laterally, these are more compact (Fig. 7d). We speculate that dense packing of fibres running to F5a may prevent adequate tracking through this region, which may explain why fibres do not project more anteriorly to reach prefrontal sectors.

## Discussion

Magnetic resonance techniques such as dMRI and resting state FC were first introduced in the late 1990s to investigate the structural and functional architecture of the central nervous system in human subjects (see Pierpaoli et al. 1996; Biswal 2012). By providing information that can be collected non-invasively in a digital format, these approaches have great utility for clinical and research purposes. Using these data to construct maps of connections, whether as tractography or functional connectivity, provides considerably opportunities for studying brain architecture and its functional role. In the animal model, especially in the macaque, detailed knowledge of cortical connectivity and functional organization of motor control has been collected based on neural tracer and electrophysiological experiments, which enables some validation to be made of these MR-based approaches and, thus, assessment of how accurate they may be in representing genuine anatomical and functional organisation. A growing number of studies have compared data on the cortical connectivity of the macaque brain with dMRI data (e.g., Markov et al. 2014; Thomas et al. 2014; van den Heuvel et al. 2015; Azadbakht et al. 2015; Donahue et al. 2016), arriving at different conclusions on the accuracy of dMRI in tracing neural connections. Other studies have used rs-FC to highlight functional correlations between cortical areas of the macaque brain which may indicate some similarity in the functional architecture of the macaque and the human brain (Hutchison et al. 2011, 2015). In the present study, we test the efficacy and accuracy of different dMRI and rs-FC techniques in identifying two large-scale cortical motor control networks (i.e., the lateral grasping network and the exploratory oculomotor network) in the living macaque brain, for which there is detailed, solid knowledge in terms of involved areas and interconnections based on neural tracing and electrophysiological studies. In contrast with other studies, the cortical sectors used as seeds have been defined in every monkey in order to include the core of each cortical area of the networks under study.

This investigation showed that dMRI and rs-FC produce different results in terms of estimating connections. Tractography was able to detect connections with higher specificity than resting state techniques, but less sensitivity. Importantly, both deterministic and probabilistic methods provided false positives and negatives and were not always in accordance as to the connections identified. Tractography has limited utility for studying short-range connections within directly adjacent cortex, although it can be used to show U-shaped fibres between adjacent gyri (Catani et al. 2012, 2017; Guevara et al. 2017). Long range fibre bundles are more reliably reproduced with tractography (e.g., Rojkova et al. 2016; Warrington et al. 2020). As such, it is noteworthy that neural tracing studies show that connections of a given cortical area typically involve, qualitatively and quantitatively, mostly adjacent cortex and neighbouring areas and that long-distance connections, although important from a functional point of view, generally represent a minor component of the total labelled cells. For example, after neural tracer injections in F5p, about 60–70% of the labelled neurons are located within the primary motor/premotor cortex, whereas only about 4–5% of the labelled cells are located in IPL areas AIP and PFG, which are sources of visual information crucial for selecting and controlling object-oriented hand actions (Gerbella et al. 2011). Thus, comparing long-distance, point-to-point cortical connectivity is somewhat challenging when comparing the accuracy of dMRI in identifying connections between different sectors. For this reason, we instead compared the presence of connections between techniques, however, this may be an area for future study. On the other hand, resting state techniques were better able to identify local over long-range functional connectivity, but their interpretation is less clear. As discussed below, the present data, in agreement with some previous studies (Thomas et al. 2014; Reveley et al. 2015), indicate some expedients that may be helpful in improving the quality of these neuroimaging techniques in representing the underlying anatomy.

### Comparing dMRI with neural tracing

In the present study, different sectors of the LGNet and EONet were defined on the cortical mantle, and for tractography analysis were slightly extended into the immediately contiguous white matter. Tractography was primarily effective in identifying fibres connecting parietal and frontal cortex, in particular those projecting from AIP to SII, F5a and F5c, although projections to prefrontal sectors could not be identified. Connections between the posterior bank of the arcuate sulcus, ventral premotor regions (F5) and rostral inferior parietal regions (PFG, PF, AIP) likely constitute the macaque homologue of the ventral branch of the superior longitudinal fasciculus (SLF III), making up the core component of the LGNet (Croxson 2005; Schmahmann et al. 2007; Mars et al. 2011; Warrington et al. 2020). Despite marked differences in gyral and areal organization, similarities in the organization of parieto-frontal connections in the macaque and the human brain have been reported (e.g., Thiebaut de Schotten et al. 2012). In the human brain, the SLF III connects the rostral IPL (supramarginal gyrus; BA40) with the ventral portion of the precentral gyrus (BA6) and the inferior frontal gyrus (BA44, 45) (Thiebaut de Schotten et al. 2011b).

On the other hand, both structural and functional connectivity techniques could not identify connections running directly between the anterior bank of the arcuate sulcus (FEF, 45B) and caudal inferior parietal regions (LIP). These fibres constitute part of the middle branch of the superior longitudinal fasciculus (SLF II), the core parieto-frontal connection of the exploratory oculomotor network (Sani et al. 2019). These fibres run in parallel but more medial to the SLF III in an anterior–posterior direction, with a clear distinction between each tract bundle. We speculate that tractography algorithms may be unable to bend and track dorsally to reach the prearcuate cortex, where the oculomotor areas FEF, 45B, and caudal 46v are located. Hence, to reproduce fibres of this tract in the macaque, regions-of-interest within deeper white matter beneath the arcuate sulcus may be required.

We compared representative trajectories of labelled axons extending from AIP with spherical deconvolution modelling used to generate tractography, to assess whether the fODF within a single voxel was reflective of likely axon trajectories. We first showed AIP-frontal projections running dorsally above SII, and tractography was able to reliably reproduce the projections that also extended into SII. We also showed that AIP-F5 connections shown with tracers could also be reproduced using tractography. However, further along this tract, within the frontal lobe, streamlines projecting through and past F5 (mainly F5a) were not connected with prefrontal sectors (12r and 46v). A previous study comparing postmortem dMRI tractography with histological analysis showed that the presence of uniform and dense sheets of fibres running below and parallel to layer VI poses challenges for diffusion tracking into sulcal regions, as well as into gyral crowns (Reveley et al. 2015). These dense U-shaped connections surround the arcuate sulcus, as well as within the parietal lobe and may hence influence the efficacy of tractography in projecting through this area (Schmahmann and Pandya 2006; Catani et al. 2012, 2017).

Previous studies, which have compared dMRI with neural tracer data in the macaque brain (Markov et al. 2014; Thomas et al. 2014; van den Heuvel et al. 2015; Azadbakht et al. 2015; Donahue et al. 2016) also show that confining regions of interest (ROIs) to gray matter strongly reduces the sensitivity of dMRI. For this reason, it is necessary to extend ROIs into the underlying white matter, as we did, however this may reduce the specificity of results. Comparisons of different dMRI analyses show that by changing parameters it is possible to alter sensitivity (measured as true positives) and specificity (measured as false positives) of results. We compared different tractography approaches, but in fact showed that there was fairly good correspondence between techniques, with only a slightly increased risk of increasing false positives when using probabilistic approaches. It has been hypothesized (van den Heuvel et al. 2015) that false positive results obtained with dMRI could also be explained by the lack of efficacy of neural tracers in identifying some connections (false negatives). We were unable to evaluate this in the present study, as we focused on identifying well-documented connections from neural tracer studies, although it may hence be important to also compare tractography techniques with other invasive approaches such as polarised light imaging (Axer et al. 2011).

Our results indicate that comparing genuine fibre trajectories, identified with neural tracing, with non-invasive approaches may help to highlight areas for which tractography lack accuracy (for example projections from AIP to prefrontal sectors). This information is relevant in improving the accuracy of tractography output (Smith et al. 2012; Jbabdi et al. 2015). For example, in human studies, this approach has improved tracking of Meyer’s loop of the optic radiation as well as the acoustic radiation, both of which are challenging to reproduce with most tractography algorithms (Chamberland et al. 2017; Maffei et al. 2018). There have been a number of recent studies showing inherent biases in tractography output, which vary depending on the acquisition, preprocessing and dissection approach used (Dyrby et al. 2011; Maier-Hein et al. 2017; Jeurissen et al. 2019). While simulated phantoms are commonly used to appraise the reliability of models and algorithms in human studies (e.g., Poupon et al. 2008; Neher et al. 2014), the availability of tracing data and the growing field of comparative MRI may provide a meaningful opportunity to identify anatomical and orientational priors to improve tractography (Rheault et al. 2019).

### Comparing resting state MRI with neural tracing

We showed that rs-FC was partially effective in identifying the various different nodes of both the LGNet and the EONet. Indeed, the rs-FC matrix in Fig. 5 shows several examples of coherent fluctuations of BOLD signals of ROIs, which are anatomically connected and are involved in the LGNet or the EONet. For example, various F5 subdivisions appeared to be functionally correlated with rostral IPL areas, area SII and the insula, and area 45B appeared to be functionally correlated with the temporal cortex.

However, the connectional matrix also shows that all the various ROIs tend to be strongly functionally correlated with adjacent cortical areas of the same or the other network. Furthermore, the maps in Fig. 5 show that the various ROIs were typically at the centre of a relatively large, fairly homogeneously extending, functionally correlated region. Thus, the FEF, for example, was functionally correlated with adjacent oculomotor prefrontal areas, but also with ventral premotor cortex and even with the primary motor area F1. Accordingly, our data provide evidence for a clear tendency of rs-FC to show false positive, short distance “connections”.

The matrix also showed relatively poor long-distance rs-FC for many ROIs. For example, area LIP did not correlate with either of the prefrontal oculomotor areas, or the temporal cortex. Accordingly, long-distance rs-FC may be affected by false negatives. This could partially be attributed to inter-individual differences in areal localization that can affect the identification, at the group level, of subtle differences in rs-FC. This was also confirmed by the results of the UC Davis individual level rs-FC analysis, which showed long-distance rs-FC that was absent at the group level (e.g., LIP-FEF and AIP-F5c). These connections may have been better identified through the use of individual definition of the intrasulcal areas.

The previously described limitations appear to be a common problem of rs-FC in macaques, even when different approaches are used (Mars et al. 2011; Neubert et al. 2014; Hutchison et al. 2015; Sharma et al. 2019). Using a different rs-FC analysis, not requiring a priori seed definition, Hutchinson and colleagues (2011) defined eleven different networks across the entire macaque cortex. In most cases, these networks included mostly neighbouring regions, rather than anatomically connected zones, as well as very few distant regions, except for homologous contralateral areas. Sharma and colleagues (2019) describe different patterns of rs-FC of F5 subdivisions using a contrast agent for enhancing signals in awake macaque monkeys. Their results appear to be very similar to those we observed. Indeed, although the observed patterns appear to vary according to the location of the seeds, all tended to involve a large cortical region around the seed, including the FEF and neighbouring prefrontal oculomotor areas.

Some similarities with our results were also observed in other studies (Mars et al. 2011; Neubert et al. 2014) in which, for example, prearcuate oculomotor areas showed rs-FC with F5 and F1 (false positive) and did not show rs-FC connectivity with inferotemporal areas (false negative). It therefore seems from the present and other studies that rs-FC shows relatively similar and reproducible patterns even when different approaches are used. Furthermore, most studies (e.g., Babapoor-Farrokhran et al. 2013; Neubert et al. 2014; Hutchison et al. 2015; Sharma et al. 2019) show specificity of these patterns, even when adjacent zones are compared. Abrupt variations in rs-FC patterns among adjacent zones have been used to define areal parcellation in the human brain (Cohen et al. 2008; Xu et al. 2019). However, this FC-based parcellation approach should be adopted with caution. Indeed, as already pointed out in several studies and in line with our data, coherent fluctuations of BOLD signal do not necessarily reflect direct cortical connectivity or common functional properties. Furthermore, the possible contribution of indirect, polysynaptic connectivity, or of common subcortical input may be variable across cases and cannot be assessed easily with this method. It is also challenging to interpret how there can be a lack of functional connectivity between areas that are relatively strongly anatomically connected. Finally, another aspect that still remains to be clarified is why rs-FC and tracer patterns appear to be in better concordance for somatomotor areas than for other regions such as, e.g., prefrontal cortex (Van Essen et al. 2019). One possible explanation could be that variability across monkeys in functional connectivity appears to be lower for the primary sensory and motor areas than the high-order association regions which make up the majority of long-distance connections (Xu et al. 2019).

One paradigmatic example, based on our data, of the possible difficulties in explaining rs-FC data in light of current interpretations is represented by the correlation between the FEF and F1, observed here and in other studies discussed above. It is well established that these two areas lack direct anatomical connections and do not appear to share common connections with other cortical areas, but also have markedly different input from the thalamus.

### Limitations

Non-human primate neuroimaging is a growing field, although its quality is not yet at the stage of human neuroimaging, as there are unique challenges to be faced when acquiring this data. High field strengths are commonly used, much higher than those regularly used for human studies (around 3T), with custom built surface coils which can result in B1 homogeneity and varying coil coverage which can cause alterations in image intensity. This also leads to distortions and dephasing due to susceptibility. As such, pipelines to process these data have to be carefully optimised, and until the release of the recent PRIME-DE resource (Milham et al. 2018, 2020), there has been no established benchmark from which to establish data quality. There were some discrepancies between the connections identified here and those described in previous studies (Warrington et al. 2020; Schmahmann et al. 2007; Sani et al. 2019), which may indicate that data quality or data processing may not have been optimised to visualise these connections. Future studies may use the sectors provided here to study different datasets, such as those acquired postmortem, to evaluate whether the connections can be identified. In terms of rs-FC analysis, it is important to note that it can be affected by state, and both cohorts of monkeys were anaesthetised (Xu et al. 2019). It also remains to establish whether rs-FC is more similar to anatomical tracing and tractography results within the awake state.

## Conclusions

In summary, the present study strongly indicates that invasive anatomical data acquired in the macaque using axonal tracing and electrophysiological approaches may provide a useful starting point for studying motor control networks using structural and functional comparative MR approaches. The two methods for mapping macaque connectomes are complementary, in that it is possible to directly validate neuroimaging data with the ‘gold standard’ showing axonal trajectories, and once these are established, to use imaging data to evaluate interindividual similarities and differences. The detailed study of the architectonics and connectional anatomy of specific circuits, such as those involved in hand and eye movements, is a basis from which it is possible to expand to study whole brain networks with comparative MR. Once these systems can be reliably reproduced, it may be possible to evaluate the emergence of uniquely human features of motor behaviour such as population-level right hand preference (Howells et al. 2018). Comparative MR is a promising and rapidly growing area of research, and we here emphasise that validation of non-invasive approaches with genuine anatomy is a priority, which will also be of great benefit to the human neuroimaging community.

## Electronic supplementary material

Below is the link to the electronic supplementary material.

Supplementary file1 (DOCX 272 kb) Supplementary Figures 1. Radar chart showing ROI size measured in voxels for each monkey in the Mount Sinai cohort. 2. Regions of interest were delineated on cortical sectors (L, left) but when used for diffusion tractography were slightly extended into the white matter as shown in (R, right).
