# Supplementary figures and images for: Reproducing macaque lateral grasping and oculomotor networks using resting state functional connectivity and diffusion tractography
Source: Brain Struct Funct. 2020 Sep 16;225(8):2533–51. doi: 10.1007/s00429-020-02142-2 (PMC7544728; doi:10.1007/s00429-020-02142-2)

**Supplementary Figures**


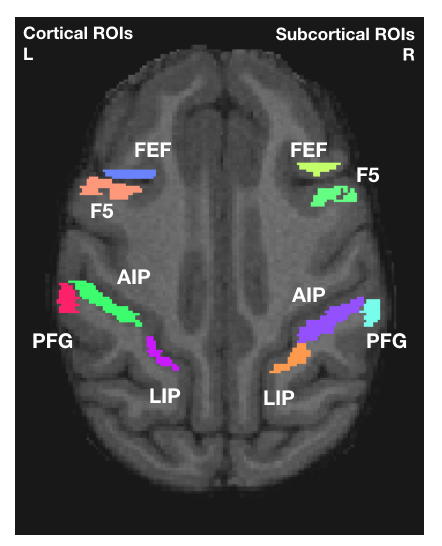


Supplementary Figure 1.


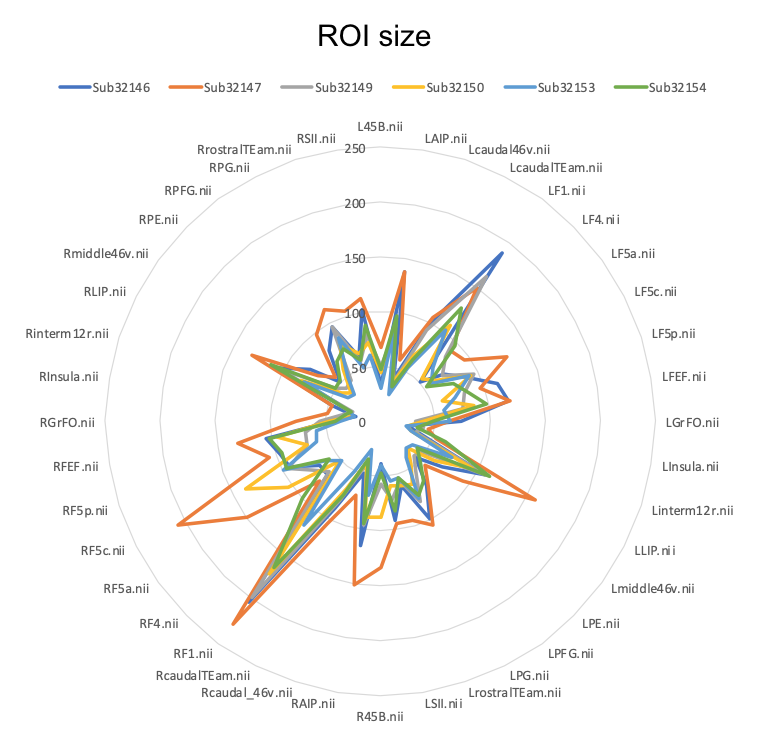


Supplementary Figure 2.

Supplement: Supplementary file 1 — Supplementary file1 (DOCX 272 kb) Supplementary Figures 1. Radar chart showing ROI size measured in voxels for each monkey in the Mount Sinai cohort. 2. Regions of interest were delineated on cortical sectors (L, left) but when used for diffusion tractography were slightly extended into the white matter as shown in (R, right). [file 429_2020_2142_MOESM1_ESM.docx]
